# Towards Sustainable Food Packaging: Mechanical Recycling Effects on Thermochromic Polymers Performance

**DOI:** 10.3390/polym17081042

**Published:** 2025-04-11

**Authors:** Colette Breheny, Declan Mary Colbert, Gilberto Bezerra, Joseph Geever, Luke M. Geever

**Affiliations:** Polymer, Recycling, Industrial, Sustainability and Manufacturing (PRISM) Research Institute, Technological University of the Shannon, University Road, N37 HD68 Athlone, Ireland; declan.colbert@tus.ie (D.M.C.); gilberto.bezerra@tus.ie (G.B.); joseph.geever@tus.ie (J.G.)

**Keywords:** thermochromic pigments, food packaging, mechanical recycling, sustainability, polymer degradation

## Abstract

Integrating thermochromic pigments (TPs) into food packaging offers significant benefits for monitoring temperature variations, improving food safety, and reducing waste. However, the recyclability of such materials remains underexplored, particularly regarding the retention of their optical and mechanical properties after repeated recycling. Addressing this gap, this research aims to evaluate how mechanical recycling affects key properties of polypropylene (PP) blends containing varying TP concentrations. Three formulations, PP100/TP0 (0% TP), PP98/TP2 (2% TP), and PP92/TP8 (8% TP), were subjected to five recycling cycles, with changes in thermal stability, color transition behavior, mechanical integrity, and surface morphology analyzed. The results indicate that PP100/TP0 maintained its mechanical integrity with minimal degradation (6% absolute crystallinity loss; color difference ΔE*_ab_ = 1.45) across recycling cycles. However, blends containing TPs exhibited progressive deterioration. P98/TP2 displayed moderate reductions in mechanical strength (−10.8%) and thermochromic efficiency (color change ΔE*_ab_ = 6.52), while PP92/TP8 showed significant degradation, including increased activation temperatures (+3.8 °C) and color vibrancy loss (42.9% loss in saturation). These effects were attributed to polymer breakdown, pigment aggregation, and altered crystallinity. Despite the limitations of recyclability, this study provides critical insights into the feasibility of TPs in sustainable, intelligent food packaging. Further research is required to enhance TP stability during reprocessing, ensuring long-term functionality in circular packaging systems.

## 1. Introduction

Material science is an interdisciplinary field that studies materials and their properties. It is a challenging and stimulating area of research characterized by rapid and numerous breakthroughs, including novel materials [1]. Among novel materials, smart polymers are gaining interest because of their capacity to respond to stimuli in their environment in a controlled manner [2]. Smart polymers can exhibit various responses, such as changes in shape, solubility, or optical properties, depending on the applied stimulus [3]. One notable example is smart polymers that undergo color changes in response to external stimuli such as pH (chemochromogenic), light (photochromogenic), temperature (thermochromogenic), and magnetic fields (magnetochromogenic) [4]. Among these, thermochromic polymers (TPs) stand out for their distinct ability to alter color in response to changes in temperature [5]. As noted by several studies, TPs are attractive to numerous applications, including food packaging [6,7,8].

Integrating thermochromic pigments (TPs) into packaging materials that meet food safety standards offers a promising solution for monitoring recommended food storage conditions throughout the supply chain [9]. Food packaging protects food from environmental elements such as oxygen, moisture, light, temperature, and microorganisms [10]. Through its ability to detect temperature changes, TP packaging thereby aids in maintaining food quality and prolonging the shelf life of the food [11]. Customer awareness surrounding food safety and the desire to reduce food waste has created a demand for intelligent food packaging [12]. By incorporating TPs into food packaging, food suppliers can reduce the likelihood of spoiled food by providing customers with vital information regarding the supply chain storage temperature of the packaged food [13].

TPs undergo a reversible phase transition at a specific temperature [14,15]. This physical change alters the material’s optical characteristics, resulting in a color change [16]. The color modification is intended at specific temperatures, such as room temperature (22 °C), refrigeration temperature (4 °C), or freezing temperature (−18 °C), indicating whether food items have been subjected to temperatures outside the permitted range [17,18,19]. Monitoring temperature fluctuations through a visual alteration of the food packaging is particularly important for perishable items, including dairy products, meats, and frozen meals, where even a short exposure to temperatures outside the recommended range can result in food decomposition [20].

While TPs offer benefits in food packaging, significant concerns arise from their environmental impact [21]. The extensive use of synthetic polymers, including thermochromic materials, has prompted environmental concerns, mainly surrounding plastic waste [22]. Plastic is one of the most extensively utilized materials in the modern world [23]. The global use of plastic materials is rising due to their durability, cost-efficiency, and lightweight properties [24]. Plastic packaging (i.e., film wraps, bags, and other packaging materials) is the primary form of plastic waste worldwide. In Europe, 39.9% of plastic is utilized for packaging [25], and single-use plastics significantly contribute to plastic pollution [26]. Disposable plastic packaging has increased due to its preservation capabilities, protective qualities, and cost-effectiveness [27].

Food packaging is expanding due to the rising global need for food driven by population increase [28]. Most food packaging is designed for on-the-go consumption and comprises plastics discarded shortly after use [29]. Plastic environmental contamination, predominantly in coastal and marine ecosystems, is recognized as a severe anthropogenic issue [30]. Microplastics, derived from the decomposition of larger plastic items, have been detected in the food chain, oceans, and rivers, endangering humans and animals [31].

In response to these environmental concerns, there is an increasing demand for recyclable, sustainable packaging materials, thereby contributing to protecting the environment [32]. Fewer than 10% of plastics produced globally are recycled; the remainder is incinerated, deposited in landfills, or released into the environment [33]. Recycling is reusing or transforming waste materials to be reused in producing new products [34]. Four main types of recycling processes exist: primary recycling (closed-loop recycling), secondary recycling (mechanical recycling), tertiary recycling (chemical recycling), and quaternary recycling (energy recovery) [35].

Mechanical recycling is a typical technique for recycling single-use plastics [36]. It involves collecting, sorting, cleaning, and reprocessing plastic waste into new products [37]. It is commonly defined as fragmenting plastics into smaller particles through shredding and shearing, then melting and blending to create a homogeneous polymer mixture [38]. Mechanical recycling mitigates plastic waste and conserves resources by reusing plastic waste that otherwise would be discarded in landfills or the natural environment [39]. However, it is challenging to recycle high-performance, multiple-functional plastics [40]. Recycling may substantially alter the properties of the recycled materials, particularly when subjected to multiple recycling cycles. These changes include reduced mechanical strength, thermal stability, and optical characteristics [41].

Numerous studies have examined the impact of mechanical recycling on polymer resins, such as polypropylene [42,43,44]. However, the effect of mechanical recycling on polymer resins infused with TPs, particularly those used in food packaging, is under-researched. This under-exploited area presents a significant gap in understanding the recycling of TPs. If temperature sensitivity is compromised, it may become impossible to properly alert customers to temperature changes in food storage [45]. If TPs are no longer capable of accurately detecting a discrete temperature change, their distinctive ability to detect and respond optically to temperature fluctuations becomes redundant, making them unsuitable for their intended use in food packaging [46].

The primary concern surrounding the mechanical recycling of TPs is the potential degradation of their optical properties [47]. Thermochromic materials exhibit color-changing characteristics due to intricate interactions between the polymer matrix and the thermochromic agent. However, this unique thermochromic property is vulnerable during the recycling process due to additional high processing temperatures and mechanical shear stresses, potentially resulting in the degradation of the thermochromic masterbatch or the formation of defects in the base polymer matrix [48].

In addition to optical properties, smart polymers’ mechanical properties are also critical [49]. Food packaging must maintain specific mechanical characteristics to protect food from physical damage during storage, transportation, and distribution [50]. Mechanical properties, such as tensile strength, elongation at break, Young’s modulus, and impact resistance, indicate a material’s performance [51]. The recycling process can reduce these properties due to the degradation of the polymer chains caused by high processing temperatures [52]. In addition, the presence of thermochromic additives blended with the base polymer could alter the crystallinity of the polymer matrix during recycling, further affecting the material’s properties [53].

It remains unknown whether recycling affects the optical and mechanical properties of TPs. Understanding the impact of recycling on the properties of TPs is central to determining the feasibility of employing TPs in food packaging applications. It could inform the development of modifications required to mitigate any potential adverse effects of recycling. To the best of our knowledge, no study has investigated this. This study addresses these research gaps by examining the impact of mechanical recycling on thermochromic food-grade polymers’ optical and mechanical properties. Color stability, tensile strength, impact resistance, and other relevant properties are evaluated through multiple recycling steps. The findings of this study will provide a comprehensive understanding of how recycling affects TPs and will offer guidance for recyclable intelligent food packaging materials.

## 2. Materials and Methods

### 2.1. Materials

A commercially available food-grade nucleated polypropylene (PP) (Moplen HP548R, Mw 26,000 g/mol, LyondellBasell, UK) was supplied by Ross Polymer (Athlone, Ireland). The material had a density of 0.9 g/cm^3^. Additionally, the manufacturer certified the material as suitable for food contact applications.

Thermochromic pigment (ThermoBatch™) obtained from SpotSee^®^/LCR Hallcrest Ltd. (Chester, UK) was supplied in pellet form with a reversible temperature transition of 41 °C. It had a density of 0.508 g/cm^3^ and an MFI range from 15 to 40 g/10 min.

Unless otherwise stated, all testing was conducted at 23 °C ± 2 °C, in accordance with the requirements of ISO 527-2:2012 [54], ISO 179-1:2023 [55], and other applicable standards. These conditions ensured consistent and reproducible results across tensile, impact, and other property evaluations.

### 2.2. Preparation of Polypropylene and Thermochromic Pigment Mixture

Binary mixtures of PP as the matrix and TP as the additive were manually dry-mixed by tumbling in a sealed polyethylene bag for five minutes to ensure homogeneity. Each blend weighed 2.5 kg. The components were accurately weighed using a Sartorius LA230P analytical balance (Sartorius, Dublin, Ireland) with a precision of ±0.0001 g. The colored concentrate ThermoBatch™ contained a compatible PP polymeric carrier miscible with the PP matrix. After blending, the samples were conditioned at 23 °C for 24 h before testing to ensure consistent results. The blend compositions are shown in Table 1.

### 2.3. Injection Molding

Injection molding is a processing technology that melts polymer material with the aid of a screw and external heating bands and then injects it into a mold to form the equivalent product as the mold cools [56]. Injection molding was carried out per ISO 294-1: 2017 [57]. The blends in Table 1 were processed using an Arburg Allrounder 370E 600 E drive injection molding machine (Arburg, Lossburg, Germany). The machine was fitted with a “two by two” family mold with a double-T runner to produce two tensile (type B1) and two impact (type A1) test specimens. The Arburg machine had a maximum clamping force of 600 kN, a screw diameter of 30 mm, and a maximum calculated stroke volume of 85 cm^3^.

The temperature distribution along the barrel was regulated by four temperature controllers, with an additional fifth controller dedicated to managing the nozzle temperature. The temperature profile began at 170 °C at the hopper and progressively increased to 210 °C at the nozzle. A cooling period of 30 s was applied, with an injection pressure of 500 bar, an injection speed of 75 mm/s, and a holding pressure of 300 bar. The mold was maintained at a constant 30 °C using a Piovan Technologies THM 120/EN temperature control unit (JL Goor, Wicklow, Ireland). The mold had a shot size of 50 g. Tensile test specimens (ISO 527-2: 2012 specimen type 1BA [54]) were molded for each blend formulation in Table 1. Charpy impact test specimens (ISO 179-1 2023: specimen type 1, direction of blow edgewise [55]) were produced and notched with a Type A V-notch where required.

### 2.4. Simulation of Mechanical Recycling

Mechanical recycling refers to operations that recover plastic solid waste via mechanical processes; thereby, the new recycled material can be converted into new plastic products, substituting for virgin polymers or a portion of virgin polymers [58]. The mechanical recycling process was simulated using a Rapid 150-21 series rotary granulator (Rapid Granulator, Bredaryd, Sweden). The granulation process yielded granules within a 3 mm to 10 mm controlled size range. After granulation, a portion of the material was reserved for subsequent characterization. The remaining granulated material underwent reprocessing through an additional injection molding cycle, followed by another granulation stage. The process of molding and granulation was repeated five times to simulate the degradation effects of repeated mechanical recycling on the polymer material. Specimens for testing were prepared after each recycling cycle, corresponding to 0×, 1×, 2×, 3×, 4×, and 5× reprocessing cycles.

### 2.5. Visual and Physical Property Evaluations

The physical appearance of granulated polymer blends and injection-molded test specimens was assessed to evaluate changes in visual characteristics across mechanical recycling cycles. High-resolution digital photographs of each sample were captured using a high-resolution DSLR camera (Canon EOS 90D) (Canon Europa, Amstelveen, The Netherlands) with controlled LED lighting (CRI > 90) to ensure accurate color reproduction. Samples were placed on a neutral, matte white background to enhance contrast. The camera was positioned at a fixed distance and angle relative to the samples to maintain image comparability. Observations focused on parameters such as color uniformity, surface texture, and visible defects (e.g., cracks, streaking, or warping).

MATLAB (version R2024a) software (The MathWorks, Inc., Natick, MA, USA) quantified changes in hue, saturation, and brightness (HSB) for the samples by converting red, green, and blue (RGB) values using the rgb2hsv function, ensuring color vibrancy and uniformity tracking. These quantitative assessments complemented visual observations, providing valuable insight into the effects of mechanical recycling on the visual and aesthetic properties of the materials.

### 2.6. Color Measurement Used

The chromatic characteristics and color stability of mechanically multiple recycled thermochromic materials were examined using a portable, handheld sphere spectrophotometer (X-Rite SP62, Grand Rapids, MI, USA). Spectrophotometry is a technique used to measure how much light a specimen absorbs or transmits at different wavelengths [59]. The spectrophotometer was calibrated with a white tile and a zero tile in sequence before taking the measurements following the SP60 Series Spectrophotometer user instructions. The color results were presented using the Commission International de l’Eclairage (CIE) chromaticity coordinate system (L*, a*, b*) [60]. In this system, the L* value represents the luminance between black and white and ranges from 0 (black) to 100 (white). The a* and b* parameters represent chromaticity without specific numerical limits. Negative a* values correspond to green, while positive a* values correspond to red. Similarly, negative b* values correspond to blue, and positive b* values to yellow [61]. The differences (ΔL*, Δa*, and Δb*) indicate the variation between the sample and standard in L*, a*, and b* values. In addition, the total color difference (ΔE*_ab_) was calculated using Equation (1).

(1) $${\Delta E}_{ab}^{*}\;=\;[{\left(\Delta L*\right)}^{2}+({\Delta a*)}^{2}+{\left(\Delta b*\right)}^{2}{]}^{1/2}$$

where

${\Delta E}_{ab}^{*}\;$ is the color difference between two colors;
ΔL* is the difference in lightness between the two colors;
Δa* is the difference in the red–green axis;
Δb* is the difference in the yellow–blue axis.

The color coordinates L*, a*, and b* were recorded according to ISO/CIE 11664-4: 2019 [62] using Oncolor™ (v.6.3.4.4 QC-Lite) software (CyberChrome Inc., Woodstock, NY, USA). Five replicates were obtained for each group of samples, and the corresponding means and standard deviations were calculated. This coordinate made it possible to determine the color difference associated with the test specimens. The distance metric, ∆E*_ab_, was obtained by following Equation (1), compared with the color coordinates of the initially recycled virgin food grade material, and used as a reference.

### 2.7. Mass Stability Assessment

Mass measurement before testing polymer samples indicates consistency and comparability in mechanical properties across tests [63]. To ensure the comparability in mechanical properties across different recycling cycles, the mass of tensile test specimens was measured using a Mettler TG50 Thermobalance (Mettler-Toledo, Columbus, OH, USA), with a precision of ±0.0001 mg before testing. Five replicate specimens were tested for each recycling cycle to provide statistically robust results. Mass measurements’ mean and standard deviation were calculated for each sample set. This approach identified potential trends in mass changes due to material degradation or other factors during the recycling process [64].

### 2.8. Melt Mass-Flow Rate (MFR) and Melt Volume-Flow Rate (MVR) Testing Procedure

The melt mass-flow rate (MFR) test measures the mass of polymer, in grams, flowing in 10 min (g/10 min) through a heated cylinder and a standard capillary under a prescribed load and temperature [65]. Testing was carried out according to ISO 1133-1: 2022 [66]. A Rosand Precision Advanced Melt Flow Rate System instrument (Labquip, Dublin, Ireland) was used, equipped with an orifice die (2.095 mm diameter, 8 mm capillary length).

Approximately 4 g of polymer pellets were weighed on an Ohaus PA512 Pioneer precision balance (Merck Life Science Limited, Wicklow, Ireland) (±0.01 g accuracy). Testing was carried out at 230 °C using a load of 2.16 kg, as specified in the matrix material data sheet. When the set temperature of the melt flow rate system stabilized for a minimum of 15 min, the material was introduced into the barrel using a funnel to aid in the filling. A packing rod compacted the polymer pellets into the barrel, thereby reducing space between pellets, eliminating air gaps, and ensuring the polymer was uniformly melted. The piston was inserted into the barrel, and the preheat timer was activated, counting the 300 s to ensure uniform melting of the polymer sample. Towards the end of the preheat, a 2.16 kg weight was placed on the piston. A piston/weight support held the piston and weights above the upper reference mark before commencing the testing. This support was removed after the preheat was completed. The piston/weight exerted pressure on the polymer, extruding it through the die. The extrudate mass was recorded.

The melt mass-flow rate (MFR), expressed in grams per 10 min, was calculated using Equation (2).

(2) $$MFR(T,m_{nom})=\frac{600\times m}{t}$$

where

T is the test temperature in degrees Celsius;
m_nom_ is the mass, in kilograms, exerting the nominal load;
600 is the factor used to convert grams per second into grams per 10 min (600 s);
m is the average mass of the cut-offs in grams;
t is the cut-off time interval in seconds.

The melt mass-flow rate (MFR) test measures the mass of the polymer, in grams, flowing in 10 min (g/10 min) through a standard capillary under a prescribed load and temperature. Testing was carried out according to ISO 1133-1: 2022 [66].

The melt volume-flow rate (MVR) was calculated from the MFR using Equation (3).

(3) $$MVR(T,m_{nom})=\frac{MFR(T,m_{nom})}{\rho }$$

where

ρ is the density of the melt in grams per cubic centimeter and is given by the material specification standard or, if not specified therein, obtained at the test temperature.

The MVR test measures the volume of the polymer, in cubic centimeters (cm^3^), that flows in 10 min (cm^3^/10 min) through a standard capillary under a prescribed load and temperature. Testing was conducted in accordance with ISO 1133-1: 2022 [66].

Each sample was tested in quintuplicate, and the mean, standard deviation (SD), and coefficient of variation (CV) for MFR and MVR were calculated to assess consistency. Post-testing, all apparatus components, including the cylinder, piston, and die, were thoroughly cleaned before repeating the testing to prevent contamination. A go/no-go gauge verified the cleanliness of the die.

### 2.9. Thermal Characterization via Differential Scanning Calorimetry

Differential scanning calorimetry (DSC) is a thermoanalytical method used to measure heat flow (change in enthalpy) as a polymer undergoes controlled temperature fluctuations that induce phase transitions [67]. Key thermal properties, including melting point (T_m_) in °C, crystallization temperature (T_cc_) in °C, enthalpy of melting (ΔH_m_) in J/g, and glass transition temperature (T_g_) in °C were analyzed using DSC as per ISO 11357-3: 2023 [68].

Thermal characterization was performed on three replicates per sample under a nitrogen atmosphere using a Netzsch Differential Scanning Calorimeter (DSC) 214 Polyma (Netzsch, Wolverhampton, UK), calibrated with indium as the reference material. Samples weighing between 8 mg and 10 mg were precisely measured on a Kern ABJ-NM/ABS-N analytical balance (Kern, Balingen, Germany) (±0.0001 g precision). The polymers were cut into small pieces to ensure uniform thermal distribution and evenly distributed to ensure optimal contact between the sample and the aluminum crucibles, which were hermetically sealed using a crimping press. An empty pan with a lid served as an inert reference.

DSC scans were conducted at a heating rate of 10 °C/min, covering a temperature range of 20 °C to 230 °C for the test specimens, which had undergone 0, 1, 2, 3, 4, and 5 recycling steps. A constant nitrogen gas flow rate of 40 mL/min was applied to purge volatiles from the sample chamber. DSC measurements were analyzed to determine the transition temperatures using Netzsch Proteus Analysis Manager Software, version 8.0.0 (Netzsch, Wolverhampton, UK).

The percentage of crystallinity (Xc) of the various blended samples was determined using Equation (4) [68].

(4) $$\chi_{C}=\frac{{\Delta H}_{f}}{{\Delta H*}_{f}}\times 100$$

where

X_c_ is the relative percentage of crystallinity;
∆H_f_ is the melting enthalpy of fusion of the specimen in joules per gram (J/g);
∆H*_f_ is the melting enthalpy of fusion of 100% crystalline PP, 207 J/g [69].

### 2.10. Tensile Strength Testing Procedure

Tensile testing of polymers is used to evaluate a polymer’s mechanical properties by applying a controlled tensile (pulling) force until the sample breaks [70]. Tensile testing was performed on tensile specimens (specimen type 1BA) per ISO 527-2: 2012 [54] to evaluate the mechanical properties of the polymer blends across multiple recycling cycles. The analysis measured Young’s modulus, maximum tensile stress, and tensile strain at break to assess structural integrity and durability. Each polymer blend, PP100/TP0, PP98/TP2, and PP92/TP8, was tested across five recycling steps (0, 1, 2, 3, 4, and 5 cycles), with five replicate specimens per cycle to ensure statistical robustness.

Tensile test analysis was conducted using an Instron 3400 tensile tester machine (Instron, Norwood, MA, USA), equipped with a 4 kN load cell and Bluehill^®^ software version 4.29 (Instron, Norwood, MA, USA) for data acquisition. Each sample was evaluated using five specimens, each with a length of 170 mm, a width of 10 ± 0.04 mm, and a thickness of 4 ± 0.4 mm. A grip gap of 25.4 mm was maintained to ensure uniform clamping. The tensile testing was conducted under quasi-static circumstances at a 10 mm/min strain rate.

### 2.11. Charpy Impact Testing Method

The Charpy test is a standardized procedure for evaluating a material’s toughness or impact strength [71]. A pendulum hammer swings to strike the specimen during the impact test, and the impact energy is measured [72]. Charpy impact testing was performed on ten specimens following ISO 179-1: 2023 [55] using a calibrated CEAST Resil 6545 5.5 Series pendulum impact testing machine (Zwick Roell, Ulm, Germany). The tests were performed using notched and unnotched specimens to investigate the influence of TPs on impact resistance. The average value of each blend was calculated.

Test specimens, measuring 12.72 mm (±0.04 mm) average thickness, were subjected to a Type A V-shaped notch with a depth of 2 mm, created using a Zwick/Roell (Zwick/Roell, Ulm, Germany) notch cutter. A 4 joules (J) hammer, mounted on a swinging pendulum with an impact velocity of 2.9 m/s, was calibrated by releasing the pendulum without specimens to establish zero pressure. Test specimens were horizontally centered, with the notch positioned maximally centrally to the arm and the notch oriented away from the pendulum. The hammer arm was released from an elevated position to strike and fracture the specimen. The consequent downward movement of the weighted pendulum with a 4 joules (J) hammer yielded the impact energy of the test specimen in J.

The impact absorption energy of each sample was directly read from the impact tester screen. The corresponding Charpy impact strength, *a_cU_*, expressed in kilojoules per square meter (kJ/m^2^), was calculated as shown in Equation (5) for unnotched samples:

(5) $$a_{cU}=\frac{Wc}{h\times b}\times 10^{3}$$

where

*Wc* is the corrected energy, in joules, absorbed by breaking the test specimen;
*h* is the thickness, in millimeters, of the test specimen;
*b* is the width, in millimeters, of the test specimen.

The corresponding Charpy impact strength, *a_cN_*, expressed in kilojoules per square meter (kJ/m^2^), was calculated as shown in Equation (6) for notched samples:

(6) $$a_{cN}=\frac{Wc}{h\times b_{N}}\times 10^{3}$$

where

*Wc* is the corrected energy, in joules, absorbed by breaking the test specimen;
*h* is the thickness, in millimeters, of the test specimen;
*b*_N_ is the width, in millimeters, of the test specimen.

### 2.12. Morphology of Fracture Surface

Fracture toughness is a critical aspect of determining the ability of a material to resist cracking, directly influencing the overall strength and structural integrity of a component [73]. Advanced optical microscopy techniques allow analysis and comprehension of polymers’ morphological and dynamic behaviors [74]. Before conducting mechanical property tests, surface micrographs of fractured specimens from PP92/TP8 were taken using a Keyence VHX-S750E free angle observation system optical microscope (Keyence (UK) Ltd., Milton Keynes, UK) at 20× and 200× magnifications for precise imaging and SEM-like 3D observation using an advanced optical shadow effect mode. PP92/TP8 was selected for fracture surface analysis as it represents the most extreme case among the tested polymer blends due to its higher TP content (8%). Increased pigment concentration has been shown to accelerate polymer degradation, influence morphological changes, and potentially weaken fracture resistance [75]. Therefore, analyzing this blend provided the most insightful data on the effects of multiple recycling cycles, particularly regarding fracture behavior and structural deformation. The imaging focused on identifying surface roughness, micro-cracking, pigment dispersion, and clustering features. These observations allowed a comparative evaluation of the external surface quality of specimens from different recycling cycles, offering a detailed understanding of the degradation mechanisms affecting PP92/TP8.

### 2.13. SEM Imaging Protocol

Scanning electron microscopy (SEM) is a characterization technique that allows high-resolution surface topography of injection-molded impact bars across multiple recycling cycles [76]. SEM gives a 3D and high-resolution photograph of nanomaterials with a high-resolution power by examining the specimen’s surface across a beam of electrons [77]. Fractured injection-molded impact bars were examined for the external surface topography and internal fractured surface morphology using a Tescan Mira SEM. (Oxford Instruments, Cambridge, UK). Before imaging, samples were attached to adhesive conducting tape on stubs. To enhance conductivity and image, a gold coating was applied using a Baltec SCD 005 sputter coater (BAL-TEC GmbH, Schalksmühle, Germany).

Imaging was conducted at five different magnifications: 50×, 100×, 300×, 1000×, and 5000×, providing an overview of surface defects, microcracks, and pigment distribution at multiple scales. SEM analysis was performed for samples subjected to 0, 1, 3, and 5 recycling cycles, allowing for a comparative evaluation of the progressive degradation trends.

In addition to qualitative imaging, MATLAB R2024a was used to extract quantitative parameters from the SEM micrographs. Crack size measurements (mean, minimum, and maximum crack length) were obtained for PP100/TP0, PP98/TP2, and PP92/TP8 at 0 and 5 recycling steps, with measurements calibrated to the SEM scale bar. Surface roughness parameters, including Ra (mean roughness) and Rz (peak-to-valley roughness), were also calculated from the high-resolution SEM images to assess topographical degradation trends.

The SEM analysis focused on identifying microstructural changes associated with mechanical recycling, particularly the effects of TP dispersion, stress concentration, and phase separation.

### 2.14. Assessment of Temperature-Sensitive Behavior

The chemical makeup, formulation, and intended use of TPs determine how sensitive they are to temperature changes [78]. These materials may have different transition temperature ranges, and prior research has revealed differences between manufacturer specs and color transitions seen in experiments. For instance, while one study reported a manufacturer-specified transition range of 47–52 °C, the most significant color change was observed between 40 and 45° [79].

A reflectivity spectroscopy system was designed to investigate the thermal and optical properties of the thermochromic polymer test specimens before and after recycling. The experimental setup included a UV-Vis spectrophotometer (Konica Minolta CM-700d Spectrophotometer, Konica Minolta, Warrington, UK) equipped with an integrating sphere to quantify changes in reflectance as a function of temperature. This setup ensured objective spectral measurements, reducing potential errors from visual observation.

To maintain a consistent and uniform temperature distribution, specimens were placed on a digitally temperature-regulated Fisherbrand™ AREX 5 digital hotplate (±0.5 °C accuracy) with a Pt100 external temperate probe (Fisher Scientific Ireland Limited, Dublin, Ireland). The temperature control system ensured stable and reproducible heating, minimizing temperature fluctuations. Temperature sensitivity testing was conducted on PP100/TP0, PP98/TP2, and PP92/TP8 tensile specimens subjected to 0, 1, 2, 3, 4, and 5 recycling steps to determine whether mechanical reprocessing affects thermochromic activation behavior.

The thermocouple was placed in the center of each test specimen to monitor real-time surface temperature during heating. In order to assess any changes in activation temperature over several recycling stages, the manufacturer-reported transition temperature of 41 °C was used as the baseline. To ensure that samples underwent a complete transition from the original color state to the activated state, surface temperatures were regulated between 36 °C and 47 °C. Spectroscopic reflectance data were collected at regular temperature intervals to quantify the shift in optical properties throughout the transition. The spectral reflectance values were analyzed at key wavelengths to capture progressive changes in pigment activation. Additionally, testing was conducted under standardized illumination conditions (D65 light source) to eliminate variations in ambient lighting that could influence color perception.

By integrating quantitative spectroscopic analysis with precise temperature control, this methodology comprehensively assesses how recycling affects the thermochromic response of polymer blends.

### 2.15. Statistical Analyzes

Systematic and consistent data collection enhanced the rigor and reliability of the quantitative data. Outliers were not removed. The sample size for each test was specified to provide clarity and reproducibility. Statistical analyses were carried out using Minitab^®^ 21.4.1 Statistical Software (Minitab, LLC, State College, PA, USA). A one-way analysis of variance (ANOVA) was calculated for triplicate (*n* = 3), quintuplicate (*n* = 5), and decuplicate (*n* = 10) measurements, and the results were shown as a mean ± standard deviation value. This ANOVA was followed by the Tukey HSD post hoc method for pairwise comparisons. The means were considered significantly different at *p* ≤ 0.05, with a confidence level of 95%. Conclusions accounted for both statistical significance and practical importance.

## 3. Results

### 3.1. Visual and Physical Property Results

**Figure 1:**
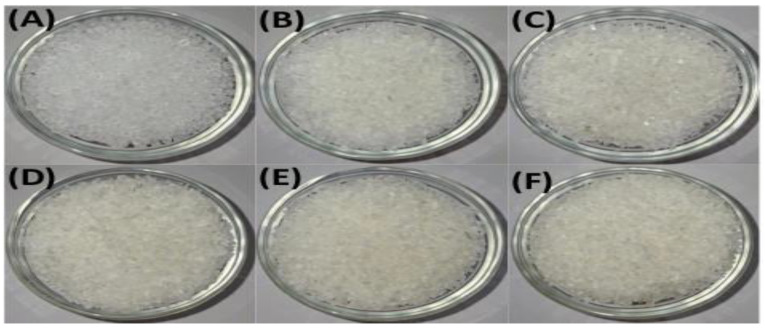
Granulated virgin PP (PP100/TP0) after 0, 1, 2, 3, 4, and 5 recycling steps: (**A**) control; (**B**) recycled × 1; (**C**) recycled × 2; (**D**) recycled × 3; (**E**) recycled × 4; (**F**) recycled × 5.

Figure 1 shows the physical appearance of the granulated virgin thermoplastic polymer blend (PP100/TP0) after zero, one, two, three, four, and five mechanical recycling iterations. Photographs were captured under controlled conditions to ensure consistency. The material retained a consistent, natural, off-white color throughout all recycling steps, indicating minimal color change.

**Figure 2:**
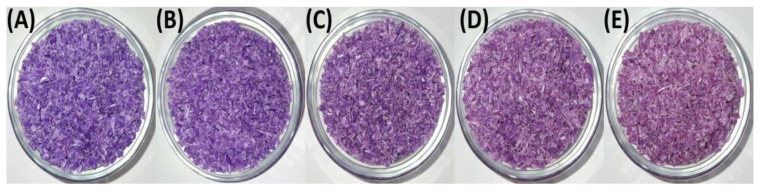
Granulated PP98/TP2 blends after 1, 2, 3, 4, and 5 recycling steps: (**A**) recycled × 1; (**B**) recycled × 2; (**C**) recycled × 3; (**D**) recycled × 4; (**E**) recycled × 5.

**Figure 3:**
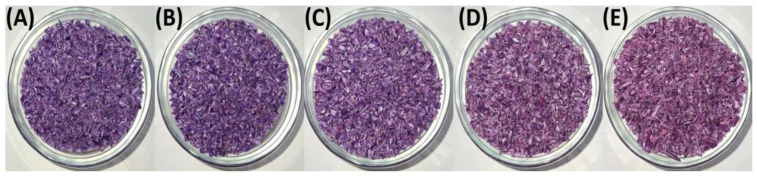
Granulated PP92/TP8 blends after 1, 2, 3, 4, and 5 recycling steps: (**A**) recycled × 1; (**B**) recycled × 2; (**C**) recycled × 3; (**D**) recycled × 4; (**E**) recycled × 5, highlighting the increasingly pale appearance of high-pigment blends due to thermochromic pigment degradation. Quantitative data extracted from these images are presented in Table 2.

Figure 2 and Figure 3 present the granulated polymer blends with 2% TP (PP98/TP2) and 8% (PP92/TP8), respectively. Both blends initially exhibited a distinctive purple hue, with the 8% TP sample showing a deeper coloration. As shown in Figure 3, the PP92/TP8 exhibited a more pronounced fading effect after five recycling cycles, suggesting pigment degradation.

**Figure 4:**
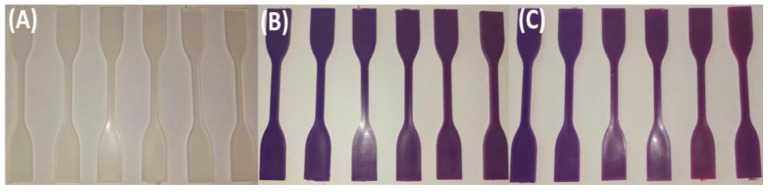
Effect of recycling on tensile test specimens of PP blends: (**A**) PP100/TP0 tensile test specimens after 0, 1, 2, 3, 4, and 5 recycling steps; (**B**) PP98/TP2 tensile test specimens after 0, 1, 2, 3, 4, and 5 recycling steps; (**C**) PP92/TP8 tensile test specimens after 0, 1, 2, 3, 4, and 5 recycling steps.

**Figure 5:**
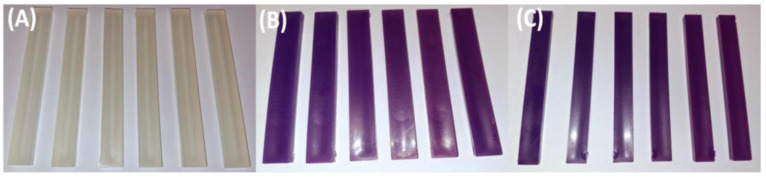
Visual comparison of impact specimens before and after recycling: (**A**) PP100/TP0 tensile test specimens after 0, 1, 2, 3, 4, and 5 recycling steps; (**B**) PP98/TP2 tensile test specimens after 0, 1, 2, 3, 4, and 5 recycling steps; (**C**) PP92/TP8 tensile test specimens after 0, 1, 2, 3, 4, and 5 recycling steps.

While Figure 1, Figure 2 and Figure 3 visually confirm progressive color fading in granulated samples, Table 2 quantifies this change using RGB and HSV color space data derived from image analysis. This combination of qualitative and quantitative assessment strengthens the interpretation of pigment degradation during recycling. Figure 4 and Figure 5 display the physical appearance of injection-molded tensile and impact test specimens with varying TP concentrations (0%, 2% TP, and 8% TP) across recycling iterations (0 to 5). Quantitative analysis confirmed that higher pigment concentrations resulted in more intense initial coloration (Table 2).

RGB values were transformed to HSV values using the digital imaging program MATLAB R2024a to analyze changes in hue and saturation during the recycling process. While visual observations provided clear evidence of color changes, quantitative analysis using RGB and HSV values was conducted to measure these variations across recycling cycles precisely. The results are summarized in Table 2. Blend PP100/TP0 provides a baseline with RGB values ranging from R: 161 to 168, G: 156 to 162, and B: 151 to 159, with HSV values showing low saturation (0.020–0.059) and high brightness (0.604–0.660), representing material with no dominant hue. In contrast, Blends PP98/2 and PP92/8 highlight progressive vibrancy loss. For instance, RGB values in PP92/8 converge to R: 153–165, G: 139–145, and B: 159–164, while HSV values show saturation dropping to 0.08–0.14 and brightness decreasing to 0.56.

These trends confirm the cumulative effects of degradation during mechanical recycling on color vibrancy. Degradation mechanisms inherent in the recycling process, such as thermal degradation [80], thermal oxidation [81], and mechanical stresses [82], are responsible for the observed color changes. Polymer chain scission, brought on by these processes, may result in altered surface characteristics [83], reduced molecular weight [84], and the formation of chromophoric groups [85].

Despite these effects, the injection-molded specimens exhibited no visible defects, such as cracks, streaks, or warping, regardless of TP concentration or the number of recycling iterations. The material’s prospective for long-term stability and performance in recycled applications is reinforced by its strong compatibility, which is consistent with independent study findings of minor structural modifications [86].

These findings highlight the progressive impact of mechanical recycling on polymer aesthetics. While PP100/TP0 retained the most consistent color, PP98/TP2 and PP92/TP8 exhibited significant fading, with hue shifting by approximately 12% and saturation decreasing by up to 35%. These changes indicate increased pigment dispersion issues, as seen in Table 2. The increasing hue shift, saturation loss, and surface defects suggest that higher pigment concentrations accelerate degradation, reducing recyclability.

### 3.2. Colorimetric Behavior of Thermochromic Polymers Post-Recycling 

Table 3 presents the color coordinates (L*, a*, b*) and the total color difference ΔE*_ab_ for thermochromic polymer specimens at each recycling step, with ΔE*_ab_ calculated relative to the unrecycled (Step 0) sample.

The near-neutral hue of PP100/TP0 is confirmed by a* and b* values remaining close to zero, indicating minimal chromaticity throughout recycling. As expected, PP100/TP0 maintained a high L* value (~71) with minimal variation throughout the five recycling steps, indicating consistent lightness and limited surface degradation. The introduction of TP masterbatch at 2% (PP98/TP2) and 8% (PP92/TP8) resulted in a significantly lower L value (~35 for PP98/TP2, ~33 for PP92/TP8), reflecting a darker appearance than the virgin polymer. This darkening effect was consistent across all recycling steps, suggesting that the TP dominates the optical properties over the degradation effects.

The a* values remained stable for PP100/TP0 (0.55–0.58), confirming no significant red–green color shift. However, PP98/TP2 and PP92/TP8 exhibited higher positive a* values (8.31–13.81), indicating a gradual shift toward the red spectrum with increased pigment concentration.

PP100/TP0 had a positive b* value (2.70–3.56), displaying a yellowish appearance that remained relatively stable during recycling. Introducing a 2% masterbatch in PP98/TP2 resulted in negative b* values (~–15), indicating a significant shift toward the blue region. PP98/TP2 and PP92/TP8 exhibited stable b* values across recycling steps, indicating that the thermochromic masterbatch largely determined the yellow-blue balance regardless of recycling.

PP100/TP0 showed low ΔE*_ab_ values (0.62–1.45), suggesting minimal perceptible color change during recycling. PP98/TP2 and PP92/TP8 exhibited significantly higher ΔE*_ab_ values (6.52), indicating more noticeable color differences than the standard. This higher color difference was attributed to the darker, bluish hue induced by the thermochromic masterbatch, which remained relatively stable across recycling steps.

A sharp decrease in ΔE*_ab_ was observed at the fifth recycling step for PP98/TP2 (79.6%) and PP92/TP8 (46.9%), indicating a substantial reduction in perceived color difference compared to previous cycles. This suggests a potential plateau or reversal in color change magnitude. Thermochromic pigment may have lost its responsive function, causing lower visual contrast and potentially muting color variation after initial pigment breakdown, aggregation, or matrix degradation. The ΔE*_ab_ values for PP100/TP0 showed a gradual increase, indicating stable optical change, indicating pigment–polymer interactions drive optical degradation rate and pattern across recycling cycles.

ANOVA confirmed that these color differences were statistically significant (*p* < 0.05), demonstrating that introducing TPs significantly altered color stability. However, Tukey’s HSD test revealed that no specific recycling step showed a statistically significant pairwise difference (*p* > 0.05), suggesting that color changes occurred progressively rather than at distinct recycling stages.

### 3.3. Mass Variation in Recycled Thermochromic Samples

Table 4 summarizes the mass measurements of tensile test specimens for the polymer blends (PP100/TP0, PP98/TP2, and PP92/TP8) across five recycling cycles. The mass values represent the mean of five replicate specimens per recycling step.

For the virgin material (PP100/TP0), an overall decrease in mass was observed, with a minor increase between Cycles 2 and 3 before continuing its downward trend. The total mass reduction over five cycles was approximately 3.2%, suggesting gradual material degradation and potential processing-related mass loss.

PP98/TP2 exhibited a similar progressive mass reduction to PP100/TP0, with both specimens experiencing progressive material loss across cycles. However, PP98/TP2 showed a slightly more significant mass reduction (4.1%) than PP100/TP0 (3.2%), suggesting minor differences in degradation behavior.

PP92/TP8 exhibited a 3.6% overall reduction in mass throughout the course of five recycling cycles. Even though there were some small variations, the average decrease was slower than that of PP98/TP2 and PP100/TP0, suggesting better mass retention during recycling.

The low standard deviation values (±0.01 to ±0.03 g) suggest that mass measurements were trustworthy and consistent among specimens, even with minor variations. ANOVA results confirmed that mass variations across recycling steps were not statistically significant (*p* > 0.05), indicating that material loss due to recycling was consistent and gradual across all specimens, without severe degradation.

The overall mass reductions observed across all specimens (3.2–4.1%) suggest that these thermochromic polymer blends retain moderate stability during mechanical recycling. The measured mass drop does not indicate material volatilization or internal chemical degradation. Instead, it likely arises from regular processing losses and does not go against the Law of Conservation of Mass. This suggests potential suitability for applications requiring multiple recycling cycles, such as secondary polymer processing or recycled material integration where slight mass loss is acceptable.

### 3.4. Melt Mass-Flow Rate (MFR) and Melt Volume-Flow Rate (MVR) Results and Interpretation

Table 5 presents the effect of multiple recycling cycles on the MFR and MVR of three polymer blends (PP100/TP0, PP98/TP2, and PP92/TP8) over five recycling steps. Additionally, the coefficients of variation (CV) for MFR and MVR are included to assess the consistency of the measurements across recycling cycles.

The MFR of PP100/TP0 increased significantly during the first recycling step (21.10–26.01 g/10 min), suggesting a significant effect of the melt flow properties and early chain scission and molecular weight reduction [87]. However, subsequent fluctuations indicate potential competing effects, such as chain recombination or oxidation stabilization [88], before a final decrease to 25.68 g/10 min at Step 5. The slower increase in MFR in later steps suggests a decreasing availability of high-molecular-weight chains, combined with possible oxidation effects that limit further chain breakage [89]. ANOVA results (*p* > 0.05) confirmed that these fluctuations were not statistically significant across recycling steps, indicating that MFR variations remain within expected process variability rather than a distinct degradation pattern.

For PP98/TP2, the MFR peaked at the second recycling step (28.54 g/10 min) before declining and stabilizing at 25.34 g/10 min by Step 5. This decrease may indicate the onset of oxidative stabilization or limited crosslinking effects, which reduce polymer mobility despite continued reprocessing [90]. However, ANOVA (*p* > 0.05) confirmed that these variations across recycling steps were not statistically significant.

PP92/TP8 exhibited an overall increase in MFR from 26.24 g/10 min to 34.94 g/10 min, indicating ongoing polymer degradation. However, MFR did not increase continuously—temporary decreases at Step 2 (–0.32 g/10 min) and Step 3 (–4.27 g/10 min) suggest non-linear degradation behavior. These fluctuations may be attributed to molecular weight redistribution or partial stabilization effects before further polymer breakdown resumes. Tukey’s HSD test revealed that MFR values for PP92/TP8 were significantly higher than those for PP100/TP0 (*p* = 0.0033) and PP98/TP2 (*p* = 0.0207), indicating that this formulation degrades at a significantly faster rate than the other specimens. However, PP100/TP0 and PP98/TP2 were statistically indistinguishable (*p* = 0.6389), indicating that the presence of 2% TP does not significantly alter MFR compared to pure PP. The presence of TPs could influence degradation by acting as a radical scavenger, temporarily slowing degradation before polymer chains undergo further breakdown [91].

The MVR for PP100/TP0 increased from 23.44 cm^3^/10 min to a peak of 29.34 cm^3^/10 min at Step 4, reflecting molecular weight reduction and improved polymer flow. However, a slight decline to 28.53 cm^3^/10 min at Step 5 suggests polymer re-entanglement or oxidative stabilization, limiting further degradation.

For PP98/TP2, MVR peaked at Step 2 (31.71 cm^3^/10 min) but declined slightly in later steps, possibly due to chain branching or crosslinking effects, which reduce polymer flow. The CV for MVR in PP98/TP2 at Step 2 (1.25%) was notably higher than in other steps, suggesting increased variability in polymer flow behavior at this stage. This may indicate instabilities in molecular weight distribution or measurement sensitivity during peak degradation. Tukey’s HSD test showed that while MVR differences between specimen types were significant, the differences between individual recycling steps within PP98/TP2 were not (*p* > 0.05), confirming that variations in MVR remain within normal process fluctuations rather than indicating a degradation threshold.

The MVR of PP92/TP8 exhibited a continuous increase, reaching 38.82 cm^3^/10 min after five recycling steps, indicating progressive chain degradation [92]. ANOVA (*p* > 0.05) indicated that these fluctuations were not statistically significant across recycling steps. Tukey’s HSD results showed that PP92/TP8 had significantly higher MVR than both PP100/TP0 (*p* = 0.0033) and PP98/TP2 (*p* = 0.0207), reinforcing that this formulation degrades faster than the other compositions.

These differing trends suggest that varying levels of thermochromic additives (TP0, TP2, and TP8) influence degradation kinetics through radical activity [93]. Some pigments may act as radical scavengers, temporarily stabilizing polymer chains, while others catalyze oxidative degradation by interacting with polymer free radicals [94]. Further analysis is required to determine whether TP in this system primarily stabilizes or accelerates degradation.

### 3.5. Thermal Transitions Observed via Differential Scanning Calorimetry

Table 6 presents the thermal properties of mechanically recycled thermochromic polymer blends over five recycling cycles. Key changes in T_g_, ΔH_cc_, T_cc_, H_m_, T_m_, and X_c_ were observed.

The glass transition temperature (T_g_) significantly declined across all polymer blends. PP100/TP0 showed the smallest decrease in T_g_, reducing from 5.0 °C to 4.1 °C (−19.78%). This suggests that the virgin polymer matrix maintained relatively stable molecular integrity. In contrast, PP92/TP8 experienced the most substantial drop in T_g_ from 4.6 °C to 2.8 °C (−48.65%), reflecting a significant loss of structural rigidity. This decrease in T_g_ suggests that polymer chain mobility increased with recycling, likely due to molecular weight reduction and structural relaxation [95]. Thermochromic pigments intensified this effect, particularly in PP92/TP8, where the highest pigment concentration led to the most significant thermal instability.

The enthalpy of cold crystallization (Hcc) decreased across all blends, indicating reduced crystallization ability with recycling. In PP100/TP0, Hcc declined from 5.0 J/g to 3.8 J/g (−24%), while PP98/TP2 showed a larger drop from 4.8 J/g to 3.5 J/g (−27%). PP92/TP8 experienced the most significant reduction, from 4.6 J/g to 2.8 J/g (−39%). This suggests that higher pigment concentrations hinder crystallization.

The cold crystallization temperature (T_cc_) followed a downward trend across all recycling cycles. In PP100/TP0, T_cc_ decreased from 110 °C to 105 °C (−4.65%). From 108 °C to 98 °C (−9.71%), PP92/TP8 showed a more noticeable decrease, indicating that the TP interfered with the crystallization process. This effect aligns with previous studies indicating that fillers and pigments can either act as nucleating agents or disrupt the crystallization process depending on their dispersion and interaction with the polymer matrix [96].

The enthalpy of melting (Hm), an indicator of crystallinity, displayed a significant decline across all blends, with PP100/TP0 reducing from 95 J/g to 83 J/g (−13.48%), PP98/TP2 decreasing from 92 J/g to 78 J/g (−16.47%), and PP92/TP8 exhibiting the most substantial drop from 88 J/g to 70 J/g (−22.79%), confirming that crystallinity loss was most pronounced in blends with higher TP concentration. The decrease in Hm suggested that crystallinity is progressively lost during recycling, and in this case, the blend with the highest pigment concentration (PP92/TP8) led to the most significant loss.

The melting temperature (T_m_) decreased with increasing recycling steps, reflecting a decline in crystalline phase stability. PP100/TP0 melting temperature (T_m_) exhibited a modest decline from 165 °C to 163.8 °C (−0.73%), whereas PP92/TP8 showed the steepest reduction, decreasing from 164 °C to 160 °C (−2.47%), suggesting that thermochromic pigment–polymer interactions weakened the crystalline phase [97]. This decline in T_m_ indicates that the thermal stability of the polymer blends decreases with recycling, particularly in blends with higher pigment content.

The crystallinity percentage (Xc) followed a similar downward trend, supporting the observed reductions in H_m_ and T_m_. PP100/TP0 retained the highest crystallinity, decreasing from 50% to 44% (−12%), while PP92/TP8 showed the most significant decline, dropping from 45% to 25% (−44%). The sharp decline in Xc for pigment-containing blends highlights the role of TPs in accelerating polymer breakdown. This effect can be attributed to pigment–polymer interactions disrupting polymer chain packing, leading to increased amorphous content [98]. ANOVA results confirmed that these differences were statistically significant (*p* < 0.05), except for PP100/TP0 (*p* > 0.05). Tukey’s HSD test further revealed that significant pairwise differences were observed at Step 4 (*p* = 0.0057) and Step 5 (*p* = 0.0156), confirming that crystallinity loss was most pronounced in later recycling cycles rather than occurring uniformly across all steps. The crystallinity decline in pigment-rich blends like PP92/TP8 is partly due to the low glass transition temperature (Tg) of thermochromic pigment’s encapsulation material. The pigment’s Tg significantly reduces the polypropylene matrix’s crystallization potential and structural order during recycling, affecting lamellar formation.

These findings indicate that thermochromic polymer blends retain some degree of thermal stability during initial recycling cycles. However, prolonged reprocessing leads to significant degradation, particularly in blends with higher pigment concentrations. The decreasing T_g_, T_cc_, T_m_, and crystallinity suggest that pigment interactions play a crucial role in determining the recyclability of thermochromic polypropylene blends. While PP100/TP0 demonstrated the highest stability across all recycling steps, PP92/TP8 exhibited the most significant deterioration, indicating that higher pigment concentrations may limit the long-term recyclability of these materials.

### 3.6. Tensile Test Results and Interpretation

Table 7 summarizes the tensile properties (Young’s modulus, maximum tensile stress, and tensile strain at break) of PP100/TP0, PP98/TP2, and PP92/TP8 across five recycling cycles.

PP100/TP0 exhibited consistent mechanical stability, maintaining a tensile modulus above 1000 MPa and tensile stress near 38 MPa throughout all recycling cycles. The strain at break indicated a slight decline in ductility over time, progressively dropping from 100.19% to 80.27% (−19.9%). ANOVA (*p* > 0.05) confirmed that these variations were not statistically significant, reinforcing that PP100/TP0 retains its mechanical integrity over multiple recycling cycles. This implies that PP100/TP0 is a good choice for food packaging that calls for long-term durability.

PP98/TP2 remained stable tensile properties through the first two recycling cycles, but a sudden strain reduction from 100.10% to 15.22% (−84.8%) at Cycle 2 is highly significant (*p* < 0.05) and suggests temporary embrittlement. ANOVA (*p* < 0.05) confirmed that tensile strain significantly changed across recycling steps, particularly in PP98/TP2, indicating an abrupt structural change. However, Tukey’s HSD test did not identify a specific recycling step with a statistically significant difference (*p* > 0.05), suggesting that the embrittlement event at Cycle 2 may be due to localized phase separation or pigment-induced structural defects rather than a distinct processing threshold [99]. However, partial strain recovery in subsequent cycles suggests chain realignment or minor recrystallization effects [100].

PP92/TP8 exhibited a progressive decrease across all cycles, with Young’s modulus decreasing from 938 MPa to 825 MPa (−12%) and tensile stress dropping from 37.55 MPa to 34.72 MPa (−7.5%) over five cycles, suggesting increasing brittleness and reduced ductility [101]. Unlike PP98/TP2, which showed some strain recovery, PP92/TP8 experienced consistent mechanical deterioration, likely accelerated by its higher pigment content (8%). The pigment may catalyze oxidative degradation or disrupt polymer chain packing, leading to faster structural breakdown [102]. ANOVA (*p* > 0.05) indicated that these changes in mechanical properties were not statistically significant across recycling steps, meaning the degradation is gradual rather than abrupt.

These results indicate a strong correlation between pigment concentration and mechanical degradation, with higher pigment levels leading to increased embrittlement [39]. PP100/TP0 displayed the highest stability, maintaining structural integrity across five cycles. PP98/TP2, on the other hand, may be restricted in high-flexibility applications due to its substantial strain variations and moderate recyclability. With the highest pigment loading, PP92/TP8 deteriorated mechanically the fastest, suggesting limited suitability for applications needing long-term durability.

These findings confirm that thermochromic polymer blends undergo mechanical degradation with increasing recycling cycles, with degradation severity increasing in blends containing higher pigment concentrations. While PP100/TP0 remains ideal for food packaging requiring multiple recycling cycles, PP98/TP2 and PP92/TP8 may be better suited for non-load-bearing applications, applications with limited recycling cycles, or where high mechanical strength is less critical.

### 3.7. Charpy Impact Test Results and Interpretation

The effect of multiple recycling cycles on the impact strength of PP100/TP0, PP98/TP2, and PP92/TP8 was evaluated using notched and unnotched samples. The results presented in Table 8 indicate that unnotched specimens remained consistently high impact strength across all recycling cycles, with an average value of 3.98 kJ/m^2^. The standard deviations showed some variations but no discernible deterioration in impact resistance due to recycling, ranging from ±1.41 to ±9.91. This stability suggests that the material can continue to absorb energy even after several reprocessing stages because no stress concentrators are present.

The presence of a notch greatly increased microstructural weaknesses, as demonstrated by the significantly decreased impact resistance of notched specimens. The impact strength values of notched samples were approximately 85% lower than those of unnotched samples in all recycling cycles. This decrease emphasizes how the notch contributes to stress localization and fracture propagation, which causes premature failure. Microstructural irregularities such as void formation, phase separation, or pigment clustering may be responsible for the increased variability in impact strength, as indicated by the rising standard deviation in subsequent recycling cycles [103].

Among the three polymer blends, PP92/TP8 displayed the highest notched impact strength among the blends, particularly in the early recycling cycles (Cycle 1—27.90 kJ/m^2^ vs. PP100/TP0: Cycle 4—25.17 kJ/m^2^). This suggests that higher TP content initially enhances impact resistance, potentially by modifying crack propagation pathways and improving fracture toughness. However, this advantage diminished as recycling progressed, indicating that while pigments may delay crack initiation, they do not prevent long-term mechanical degradation. After multiple cycles, PP92/TP8’s notched impact strength decline suggests that TPs may introduce additional stress concentration points, accelerating polymer embrittlement in later recycling stages.

Impact resistance is critical in food packaging applications, particularly for materials subjected to repeated mechanical stresses, such as rigid containers, closures, and lids. The significant reduction in impact strength for notched samples suggests that recycled versions of these polymers may be more susceptible to cracking or failure in areas with pre-existing flaws or stress concentration points. For applications that require high durability and resistance to impact loading, polymer blends with superior notched impact strength, such as PP92/TP8 in early cycles, may be preferable. However, material selection must consider the gradual decline in impact resistance with repeated reprocessing for long-term structural integrity.

Overall, these findings emphasize the importance of stress concentration in recycled polymer performance, with unnotched specimens demonstrating strong recyclability while notched specimens show increasing fragility with recycling cycles. The impact strength, both notched and unnotched, did not show any statistically significant differences across recycling steps, as indicated by the ANOVA results (*p* > 0.05). While PP92/TP8 offers better initial fracture resistance, its decline over time suggests that pigment incorporation may contribute to long-term degradation. These results underscore the need to carefully consider polymer formulation and application-specific performance requirements when using recycled thermochromic polymer blends.

### 3.8. Morphological Changes in Fractured Thermochromic Blends

**Figure 6:**
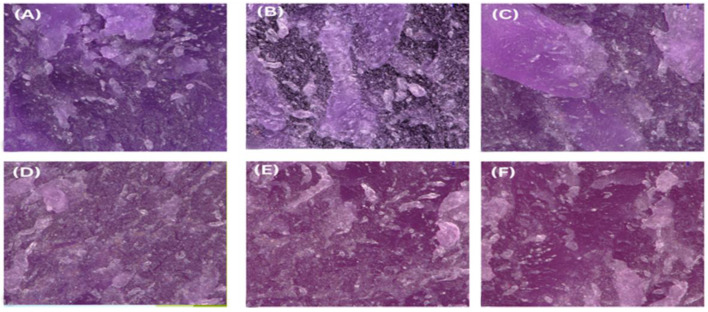
Optical microscope images of PP92/TP8 at 200× magnification using the VHX-S750E optical microscope: (**A**) 0 recycling step; (**B**) 1 recycling step; (**C**) 2 recycling steps; (**D**) 3 recycling steps; (**E**) 4 recycling steps; (**F**) 5 recycling steps.

The fracture surface morphology was examined using a Keyence VHX-S750E optical microscope at zoom 20× and 200×. Figure 6 presents optical microscopy images of the PP92/TP8 fracture surface at 200× magnification across six recycling stages. These images revealed progressive structural deterioration with increasing recycling cycles.

Heightened surface roughness (Figure 6E, seen throughout the surface, particularly in the center and lower regions), microcracking (Figure 6F, seen in the center and upper right areas), and pigment dispersion issues (Figure 6C, seen along the upper and lower margins) were among the most significant findings. The specimen surface exhibited uniform integrity with minimal imperfections at zero recycling steps (Figure 6A, smooth and evenly distributed across the field). After five recycling steps, the surface showed pronounced cracking (Figure 6F, concentrated in mid-upper-right zones) and irregular surface morphology (Figure 6E, particularly in the central region). One of these irregularities included noticeable pigment clustering (Figure 6F, observed near the right-central region). The irregularity in color uniformity and brightness, especially in the final stages, indicates degradation of the thermochromic pigment (TP), likely caused by cumulative thermal and mechanical stresses during repeated recycling [104].

### 3.9. SEM-Based Analysis of Morphological Degradation

The injection-molded impact bars’ fracture morphology and surface topography were analyzed using SEM at 50×, 100×, 300×, 1000×, and 5000× magnifications after 0, 1, 3, and 5 recycling cycles. The SEM images revealed progressive microstructural degradation, with increasing roughness, crack formation, and pigment clustering in PP98/TP2 and PP92/TP8, while PP100/TP0 remained relatively stable over multiple recycling cycles.

**Figure 7:**
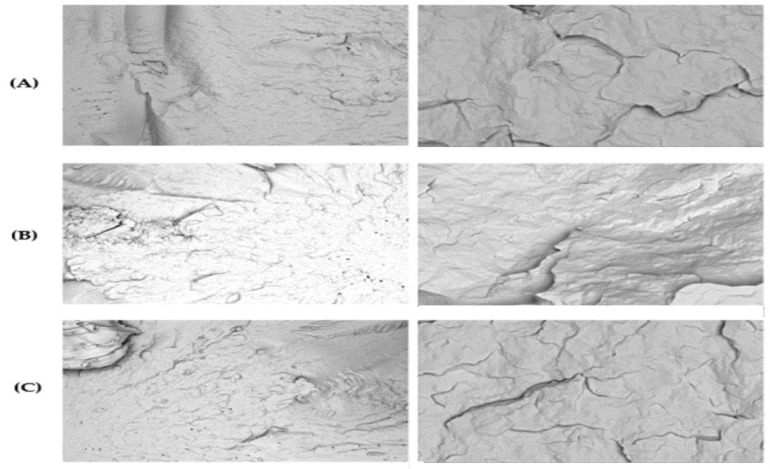
Scanning electron microscope (SEM) micrographs of PP100/TP0: (L) 50× magnification; (R) 5000× magnification. (**A**) 0 recycling steps; (**B**) 1 recycling steps; (**C**) 5 recycling steps.

As shown in Figure 7, PP100/TP0 exhibited minimal signs of degradation across all recycling cycles at 50× and 5000× magnification. In repeated recycling applications, the virgin polymer blend’s structural stability was confirmed by the smooth fracture surfaces that showed no discernible microcracks or pigment clustering. Given its constant morphology, PP100/TP0 is a suitable choice for high-durability applications, suggesting that it maintains its mechanical integrity across several recycling cycles.

**Figure 8:**
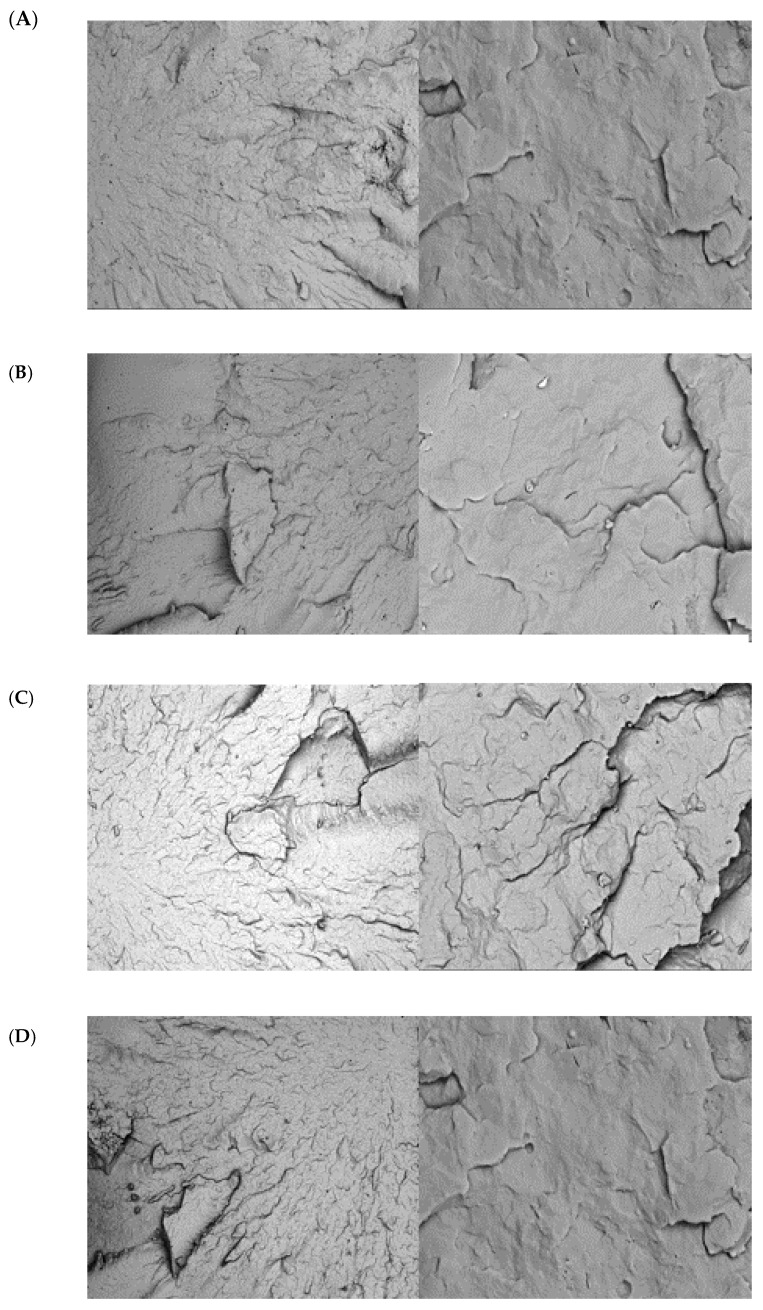
SEM micrographs of PP98/TP2: (L) 50× magnification; (R) 5000× magnification. (**A**) 0 recycling steps; (**B**) 1 recycling steps; (**C**) 3 recycling steps; (**D**) 5 recycling steps.

In contrast, PP98/TP2 (Figure 8) displayed moderate degradation, with noticeable surface roughness and pigment clustering emerging after three recycling cycles. Localized microcracks and roughened fracture edges were observed, suggesting early mechanical weakening. While the material remained suitable for limited-use applications, such as single-use trays or non-load-bearing plastic components, its progressive embrittlement beyond two cycles may reduce long-term structural performance.

**Figure 9:**
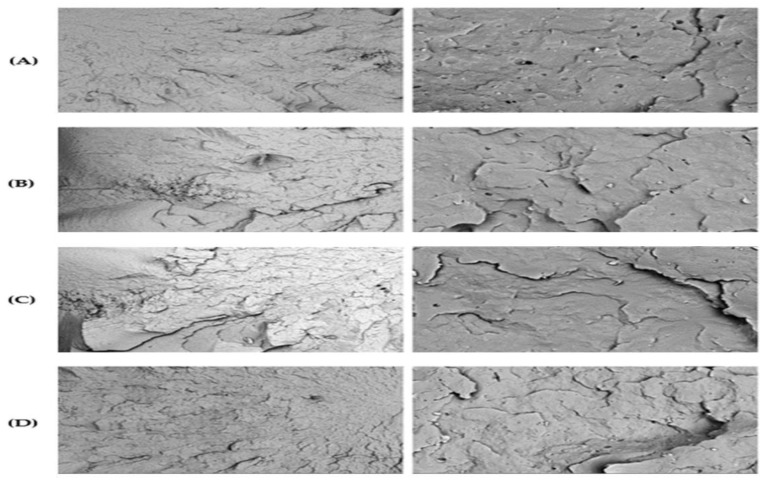
SEM images of the impact fracture of PP92/TP8: (L) 50× magnification; (R) 5000× magnification. (**A**) 0 recycling step; (**B**) 1 recycling step; (**C**) 3 recycling steps; (**D**) 5 recycling steps.

PP92/TP8 (Figure 9) exhibited the most severe degradation, with extensive roughness, widespread cracking, and pigment clustering as early as two recycling cycles. The uneven dispersion of TPs acted as stress concentration points, accelerating crack initiation and propagation, leading to brittle failure and mechanical instability [105]. The rapid degradation suggests that PP92/TP8 may not be suitable for load-bearing or high-durability applications but could still be utilized in single-use or minimally recycled products.

Table 9 illustrates the progression of crack sizes. The minimal crack growth seen in PP100/TP0, which increased from 0.2 µm (0 cycles) to 0.8 µm (5 cycles), demonstrated that the chain integrity of the polymer was well-preserved. Moderate crack propagation was seen in PP98/TP2, with mean crack size rising from 0.3 µm to 1.2 µm, suggesting increased stress concentration effects and eventual polymer degradation. The most significant increase was observed in PP92/TP8, where mean crack length increased from 0.4 µm to 2.5 µm, with a maximum crack length reaching 4.0 µm, confirming that pigment clustering and polymer instability accelerated fracture formation.

These results are corroborated by surface roughness analysis (Table 10), which demonstrates that PP100/TP0 maintained a smooth surface with Ra rising marginally from 0.05 µm to 0.15 µm across five recycling cycles, suggesting slight surface deterioration. Ra rose from 0.08 µm to 0.30 µm in PP98/TP2, indicating significant roughness growth due to increased surface wear and microstructural weakening. PP92/TP8 showed the most significant roughness increase, with Ra rising from 0.10 µm to 0.50 µm, suggesting substantial topographical degradation. The increase in Rz (maximum peak-to-valley roughness) in PP92/TP8 (from 0.25 µm to 1.00 µm) indicates the formation of deep surface irregularities, which could compromise coating adhesion and mechanical performance in applications requiring high surface integrity [105].

These findings highlight the cumulative impact of mechanical recycling on polymer microstructure. Because of its susceptibility to fracture and pigment-related stress concentrations, PP92/TP8 displayed the most severe degradation. PP98/TP2 and PP92/TP8 experienced growing microstructural degradation, which restricts their applicability for long-term use in applications needing strong mechanical resilience. They suffer growing microstructural degradation, which restricts their applicability for long-term use in applications needing strong mechanical resilience, whereas PP100/TP0 stays stable after several cycles.

### 3.10. Stability of Temperature-Sensitive Functionality Post-Recycling

The thermochromic polymer specimens, PP100/TP0, PP98/TP2, and PP92/TP8, exhibited progressive and reversible color transitions over multiple recycling cycles, with increasing recycling affecting the efficiency and responsiveness of thermochromic activation. At temperatures lower than 38 °C, all specimens maintained their original color condition with no spectrum fluctuation, according to the temperature-dependent reflectivity data, indicating no premature activation. As the surface temperature exceeded 38 °C, an increase in reflectivity and a reduction in visible spectrum absorption indicated that the TPs were entering their activation phase. Table 11 summarizes the manufacturer-specified and observed thermochromic transition temperatures of five test samples for each polymer blend before and after five recycling cycles.

PP98/TP2 showed moderate degradation, shifting its transition range from 39.1–45 °C (no cycles) to 42.3–47.1 °C (five cycles). PP98/TP2 remained functionally thermochromic, with the transition range increasing from 37.4–44.2° C (no cycles) to 42–47 °C (five cycles). Due to the strong impact of polymer breakdown and pigment instability on its heat sensitivity, the increased degradation rate raises the possibility that PP92/TP8 may not be appropriate for applications needing extended recyclability.

Recycling increased the activation temperature across all specimens, with PP100/TP0 maintaining the highest stability, PP98/TP2 experiencing moderate degradation, and PP92/TP8 showing severe optical deterioration. Full-color transition was achieved at approximately 47 °C, beyond which no further spectral changes were observed, indicating that the pigment had reached its final activated state. Higher temperatures (42.6–47.3 °C) were required to complete the color transition in samples that had undergone five recycling cycles due to delayed activation. This implies that recycling lowers pigment sensitivity, likely due to pigment clustering, oxidative instability, or polymer breakdown. As polymer degradation progresses, the TP may experience reduced dispersion efficiency, leading to inconsistencies in activation temperature and decreased contrast between color states [79].

These findings highlight the progressive impact of mechanical recycling on thermochromic efficiency, reinforcing that higher pigment loading (PP92/TP8) accelerates optical deterioration, whereas pigment-free or lower-loaded blends (PP98/TP2) exhibit better thermochromic stability over multiple recycling cycles.

## 4. Conclusions

This study highlighted the feasibility of using TPs in developing innovative smart food packaging. An evaluation of the effects of mechanical recycling on the optical and mechanical properties of PP blends containing TP was undertaken. Significant information about the stability and functionality of food-grade thermochromic polymer blends in environmentally friendly food packaging applications was obtained by subjecting them to five recycling cycles.

The results showed that materials devoid of TP were the most recyclable, retaining their mechanical integrity with minimal changes to their thermal and optical characteristics. This blend was the best option for several recycling cycles, demonstrating the least deterioration in tensile strength, impact resistance, and thermochromic activation. In contrast, PP98/TP2 displayed moderate deterioration, with increased polymer brittleness and pigment clustering observed after three recycling cycles. The thermochromic response of this blend was still functional but showed a shift in activation temperature and reduced color vibrancy, suggesting limited recyclability for applications requiring precise thermochromic control.

The degradation was especially apparent in PP92/TP8, where high pigment concentrations greatly impacted mechanical stability, optical responsiveness, and thermal behavior. Severe pigment clustering and microcracking were found by SEM analysis, which resulted in the polymer matrix’s embrittlement and decreased impact resistance.

A notable rise in activation temperature and apparent color fading rendered TPs unsuitable for extended recyclability in food packaging applications.

Thermal analysis using differential scanning calorimetry (DSC) suggested that all TP blends exhibited progressive reductions in crystallinity, glass transition temperature (T_g_), and melting enthalpy (H_m_) with increasing recycling steps. However, the degradation rate correlated with TP concentration, reinforcing that TPs influence the polymer’s long-term recyclability. The temperature sensitivity of the blends changed with recycling cycles, where higher temperatures were required for complete thermochromic transitions, especially in PP92/TP8. This effect was attributed to polymer degradation, oxidation, and pigment agglomeration, which hindered the pigments’ responsiveness to thermal stimuli.

Considering all factors, these results show how thermochromic functionality and recyclability are traded off in PP-based food packaging materials. Although TPs benefit food safety and real-time temperature monitoring, their deterioration limits long-term use. Further research is required to consider stabilizing techniques, alternative pigment blends, or production adjustments to enhance the recyclability of thermochromic polymers in food packaging. These revelations support the continuous efforts to develop sustainable, intelligent packaging solutions that balance functionality, durability, and environmental responsibility.

Even though this work was carried out under controlled conditions with specific TP concentrations, separating thermochromic packaging from regular polypropylene trash is unlikely to occur in real-world recycling circumstances. Materials that include TPs could be trace pollutants in the recycling stream. Therefore, future research should examine how low concentrations of residual thermochromic pigments (e.g., 1–2%) may affect the quality, appearance, and thermal stability of standard recycled PP, ensuring that intelligent packaging solutions do not interfere with broader recycling systems.

## Figures and Tables

**Table 1:** Blend compositions prepared for this study.

Specimen ID.	PP (wt. %)	TP (wt. %)
PP100/TP0	100	0
PP98/TP2	98	2
PP92/TP8	92	8

**Table 2:** Quantitative color data summary of blends PP100/TP0, PP98/TP2, and PP92/TP8: RGB and HSV across recycling stages.

Specimen ID.	Red (R)	Green (G)	Blue (B)	Hue (°)	Saturation (S)	Brightness (V)
PP100/TP0	161–168	156–162	151–159	Neutral	0.020–0.059	0.604–0.660
PP98/TP2	150–165	136–147	156–169	270–301	0.137–0.202	0.620–0.660
PP92/TP8	153–165	139–145	159–164	278–310	0.080–0.140	0.560–0.640

**Table 3:** L*, a*, b* color parameters and total color difference (ΔE*_ab_) across recycling steps.

Specimen ID.	No. of Recycling Steps	*L* *	*a* *	*b* *	(ΔE*_ab_)
PP100/TP0	0	71.85 (±0.04)	0.53 (±0.01)	2.41 (±0.04)	0.00
PP100/TP0	1	71.39 (±0.06)	0.58 (±0.01)	2.70 (±0.07)	0.62 (±0.09)
PP100/TP0	2	71.80 (±0.11)	0.55 (±0.02)	2.99 (±0.08)	1.09 (±0.09)
PP100/TP0	3	71.79 (±0.14)	0.57 (±0.02)	3.18 (±0.05)	1.24 (±0.10)
PP100/TP0	4	71.66 (±0.07)	0.55 (±0.02)	3.37 (±0.05)	1.34 (±0.05)
PP100/TP0	5	71.34 (±0.19)	0.55 (±0.02)	3.56 (±0.14)	1.45 (±0.10)
PP98/TP2	0	35.10 (±0.07)	10.20 (±0.04)	−15.50 (±0.11)	0.00
PP98/TP2	1	35.73 (±0.13)	10.46 (±0.09)	−15.91 (±0.17)	1.72 (±0.10)
PP98/TP2	2	35.92 (±0.09)	10.92 (±0.12)	−15.22 (±0.20)	2.45 (±0.03)
PP98/TP2	3	36.37 (±0.09)	12.52 (±0.10)	−13.68 (±0.20)	4.62 (±0.14)
PP98/TP2	4	37.21 (±0.35)	13.81 (±0.32)	−12.58 (±0.17)	6.52 (±0.24)
PP98/TP2	5	35.17 (±0.13)	10.37 (±0.31)	−16.08 (±0.42)	1.33 (±0.11)
PP92/TP8	0	34.03 (±0.11)	8.04 (±0.07)	−15.11 (±0.16)	0.00
PP92/TP8	1	33.39 (±0.14)	8.31 (±0.08)	−15.33 (±0.18)	0.80 (±0.13)
PP92/TP8	2	34.18 (±0.21)	9.69 (±0.11)	−14.80 (±0.31)	2.28 (±0.21)
PP92/TP8	3	34.53 (±0.14)	10.99 (±0.07)	−14.22 (±0.17)	3.68 (±0.04)
PP92/TP8	4	35.21 (±0.48)	12.93 (±0.51)	−12.67 (±0.78)	6.18 (±0.09)
PP92/TP8	5	34.04 (±0.36)	10.07 (±0.12)	−16.19 (±0.20)	3.28 (±0.08)

**Table 4:** The effect of multiple recycling on the mass of PP100/TP0, PP98/TP2, and PP92/TP8 after 0, 1, 2, 3, 4, and 5 recycling steps.

Specimen ID.	Recycling Steps	Mass (g)
PP100/TP0	0	3.724 (±0.01)
	1	3.712 (±0.01)
	2	3.675 (±0.03)
	3	3.696 (±0.01)
	4	3.684 (±0.01)
	5	3.606 (±0.01)
PP98/TP2	0	3.761 (±0.01)
	1	3.692 (±0.01)
	2	3.691 (±0.01)
	3	3.706 (±0.01)
	4	3.687 (±0.01)
	5	3.608 (±0.02)
PP92/TP8	0	3.763 (±0.03)
	1	3.715 (±0.01)
	2	3.722 (±0.01)
	3	3.721 (±0.01)
	4	3.699 (±0.01)
	5	3.626 (±0.02)

**Table 5:** The effect of multiple recycling on the melt flow rate (MFR) and melt volume rate (MVR) of PP100/TP0, PP98/TP2, and PP92/TP8.

Specimen ID.	Recycling Steps	MFR (g/10 min)	MFR CV (%)	MVR (cm^3^/10 min)	MVR CV (%)
PP100/TP0	0	21.10 (±0.01)	0.07	23.44 (±0.01)	0.04
	1	26.01 (±0.06)	0.23	28.90 (±0.06)	0.21
	2	23.89 (±0.03)	0.14	26.54 (±0.03)	0.11
	3	24.74 (±0.06)	0.24	27.49 (±0.06)	0.22
	4	26.41 (±0.01)	0.05	29.34 (±0.01)	0.03
	5	25.68 (±0.03)	0.13	28.53 (±0.03)	0.11
PP98/TP2	0	24.55 (±0.01)	0.04	27.28 (±0.01)	0.11
	1	24.48 (±0.34)	1.40	27.20 (±0.34)	0.04
	2	28.54 (±0.10)	0.33	31.71 (±0.10)	1.25
	3	25.31 (±0.04)	0.13	28.12 (±0.04)	0.32
	4	27.12 (±0.04)	0.15	30.13 (±0.04)	0.14
	5	25.34 (±0.08)	0.33	28.16 (±0.08)	0.28
PP92/TP8	0	26.24 (±0.01)	0.04	29.16 (±0.01)	0.13
	1	31.50 (±0.01)	0.04	35.00 (±0.01)	0.28
	2	31.18 (±0.01)	0.04	34.64 (±0.01)	0.03
	3	26.91 (±0.03)	0.13	29.90 (±0.03)	0.03
	4	29.60 (±0.04)	0.13	32.89 (±0.04)	0.03
	5	34.94 (±0.11)	0.31	38.82 (±0.11)	0.28

**Table 6:** Thermal properties observed via DSC of PP100/TP0, PP98/TP2, and PP92/TP8 after 0, 1, 2, 3, 4, and 5 recycling steps.

Specimen ID.	Recycling Steps	T_g_ (°C)	ΔH_cc_ (J/g)	T_cc_ (°C)	H_m_ (J/g)	T_m_ (°C)	X_c_ (%)
PP100/TP0	0	5.0 (±0.02)	5.0 (±0.10)	110 (±0.5)	95 (±1.2)	165.0 (±0.3)	50 (±0.8)
	1	4.9 (±0.03)	4.8 (±0.12)	109 (±0.4)	93 (±1.1)	164.8 (±0.2)	48 (±0.6)
	2	4.7 (±0.02)	4.5 (±0.15)	108 (±0.5)	90 (±1.3)	164.5 (±0.3)	47 (±0.7)
	3	4.5 (±0.03)	4.3 (±0.14)	107 (±0.4)	88 (±1.2)	164.3 (±0.2)	46 (±0.6)
	4	4.3 (±0.02)	4.0 (±0.11)	106 (±0.5)	85 (±1.4)	164.0 (±0.3)	45 (±0.7)
	5	4.1 (±0.03)	3.8 (±0.13)	105 (±0.4)	83 (±1.1)	163.8 (±0.2)	44 (±0.6)
PP98/TP2	0	4.80 (±0.02)	4.80 (±0.12)	109 (±0.5)	92 (±1.3)	164.5 (±0.3)	48 (±0.7)
	1	4.60 (±0.03)	4.50 (±0.14)	107 (±0.4)	89 (±1.2)	164.2 (±0.2)	45 (±0.6)
	2	4.30 (±0.02)	4.20 (±0.15)	105 (±0.5)	86 (±1.4)	163.8 (±0.3)	42 (±0.7)
	3	4.00 (±0.03)	4.00 (±0.13)	104 (±0.4)	83 (±1.1)	163.5 (±0.2)	40 (±0.6)
	4	3.80 (±0.02)	3.80 (±0.12)	103 (±0.5)	80 (±1.3)	163.0 (±0.3)	38 (±0.7)
	5	3.50 (±0.03)	3.50 (±0.14)	101 (±0.4)	78 (±1.2)	162.5 (±0.2)	35 (±0.6)
PP92/TP8	0	4.60 (±0.02)	4.60 (±0.12)	108 (±0.5)	88 (±1.3)	164.0 (±0.3)	45 (±0.7)
	1	4.20 (±0.03)	4.20 (±0.14)	106 (±0.4)	84 (±1.2)	163.5 (±0.2)	40 (±0.6)
	2	3.80 (±0.02)	3.80 (±0.15)	104 (±0.5)	80 (±1.4)	163.0 (±0.3)	35 (±0.7)
	3	3.50 (±0.03)	3.50 (±0.14)	102 (±0.4)	76 (±1.1)	162.0 (±0.2)	30 (±0.6)
	4	3.20 (±0.02)	3.20 (±0.12)	100 (±0.5)	72 (±1.3)	161.0 (±0.3)	28 (±0.7)
	5	2.80 (±0.03)	2.80 (±0.13)	98 (±0.4)	70 (±1.2)	160.0 (±0.2)	25 (±0.6)

**Table 7:** The effect of multiple recycling on the tensile strength of PP100/TP0, PP98/TP2, and PP92/TP8.

Specimen ID.	Recycling Steps	Youngs Modulus (MPa)	Maximum Tensile Stress (MPa)	Tensile Strain (%)
PP100/TP0	0	1016.70 (±16.02)	38.78 (±1.40)	100.19 (±8.13)
	1	1031.63 (±18.65)	38.80 (±1.32)	95.50 (±10.43)
	2	1043.69 (±18.52)	39.52 (±1.48)	90.60 (±9.76)
	3	1058.45 (±16.98)	42.30 (±1.54)	85.22 (±8.23)
	4	1049.29 (±17.45)	38.70 (±1.13)	80.27 (±7.49)
	5	1023.31 (±18.23)	38.97 (±1.23)	100.10 (±7.98)
PP98/TP2	0	1025.68 (±17.63)	39.71 (±2.53)	100.10 (±8.45)
	1	1018.90 (±19.74)	40.20 (±2.42)	95.26 (±12.97)
	2	1003.21 (±19.46)	37.95 (±2.65)	15.22 (±11.96)
	3	994.77 (±17.47)	37.83 (±2.98)	85.39 (±10.45)
	4	1001.70 (±19.91)	38.81 (±2.76)	80.62 (±10.91)
	5	857.86 (±19.95)	35.63 (±2.78)	100.26 (±10.32)
PP92/TP8	0	938.28 (±18.41)	37.55 (±3.20)	98.01 (±11.43)
	1	882.69 (±20.39)	36.54 (±3.79)	99.41 (±13.65)
	2	836.60 (±20.91)	36.92 (±3.96)	99.02 (±14.70)
	3	908.51 (±18.87)	35.82 (±4.30)	98.78 (±11.45)
	4	904.60 (±19.81)	36.04 (±3.66)	97.84 (±9.47)
	5	825.67 (±20.36)	34.72 (±3.98)	100.15 (±13.98)

**Table 8:** The effect of multiple recycling on the impact strength of PP100/TP0, PP98/TP2, and PP92/TP8 with and without notch after 0, 1, 2, 3, 4, and 5 recycling steps.

Specimen ID.	Recycling Steps	Impact Strength (kJ/m^2^) Unnotched	Impact Strength (kJ/m^2^) Notched
PP100/TP0	0	99.50 (±1.41)	15.48 (±2.56)
	1	99.35 (±1.63)	22.50 (±3.32)
	2	99.46 (±1.65)	23.95 (±4.40)
	3	99.50 (±1.76)	23.43 (±5.11)
	4	99.36 (±1.68)	25.17 (±5.93)
	5	99.55 (±1.81)	24.96 (±6.14)
PP98/TP2	0	99.55 (±1.65)	18.86 (±2.78)
	1	99.46 (±1.84)	20.25 (±3.45)
	2	99.46 (±1.83)	17.86 (±4.11)
	3	99.55 (±1.99)	20.83 (±4.04)
	4	99.55 (±2.05)	24.30 (±6.11)
	5	99.50 (±2.13)	22.20 (±7.18)
PP92/TP8	0	99.50 (±4.59)	20.90 (±7.34)
	1	99.50 (±5.27)	27.90 (±9.11)
	2	99.50 (±7.46)	19.43 (±10.00)
	3	99.50 (±8.81)	24.65 (±12.48)
	4	99.60 (±9.91)	23.03 (±13.75)
	5	99.55 (±9.12)	24.03 (±14.98)

**Table 9:** Crack size analysis across blends of PP100/TP0, PP98/TP2, and PP92/TP8 after 0 and 5 recycling steps.

Specimen ID.	Recycling Step	Mean Crack Length (µm)	Min Crack Length (µm)	Max Crack Length (µm)
PP100/TP0	0	0.2	0.1	0.5
PP100/TP0	5	0.8	0.4	1.5
PP98/TP2	0	0.3	0.2	0.7
PP98/TP2	5	1.2	0.6	2.0
PP92/TP8	0	0.4	0.3	1.0
PP92/TP8	5	2.5	1.5	4.0

**Table 10:** Surface roughness metrics across blends of PP100/TP0, PP98/TP2, and PP92/TP8 after 0 and 5 recycling steps.

Specimen ID.	Recycling Step	Ra (Mean Roughness, µm)	Rz (Max Peak-to-Valley, µm)
PP100/TP0	0	0.05	0.12
PP100/TP0	5	0.15	0.32
PP98/TP2	0	0.08	0.20
PP98/TP2	5	0.30	0.60
PP92/TP8	0	0.10	0.25
PP92/TP8	5	0.50	1.00

**Table 11:** Thermochromic transition temperatures across five samples of blends of PP100/TP0, PP98/TP2, and PP92/TP8 before and after five recycling steps.

Specimen ID.	Recycling Step	Manufacturer Transition (°C)	Observed Transition (°C)
PP98/TP2	0	41.0	39.1, 41.3, 42.0, 44.2, 45.0
PP98/TP2	5	41.0	42.3, 43.6, 43.8, 46.9, 47.1
PP92/TP8	0	41.0	37.4, 38.9, 42.1, 43.8. 44.2
PP92/TP8	5	41.0	42.6, 43.6, 45.2, 46.6, 47.3

## Data Availability

Data are contained within the article.
